# Supplementary material for: Roles of High Osmolarity Glycerol and Cell Wall Integrity Pathways in Cadmium Toxicity in Saccharomyces cerevisiae
Source: Int J Mol Sci. 2021 Jun 8;22(12):6169. doi: 10.3390/ijms22126169 (PMC8226467; doi:10.3390/ijms22126169)
Supplement: Supplementary file 1 [file ijms-22-06169-s001.zip › ijms-1231017-SI/Supplementary Table 1.pdf]

Table S1. Primers used in this study

Name	Sequence (5′-3′)
Hog1-CF	ttacggactacggtaaccaggccatacagtacgctaatgagttccaacag CGGATCCCCGGGTTAATTAA
Hog1-CR	aaaacagaaaaaaaatcatgatcgaaatacgtccactttactttgtaattg GAATTCGAGCTCGTTTAAAC
Slt2-CF	ttctagaccttgaaaaagagctggagtttggattagatagaaaatatttt CGGATCCCCGGGTTAATTAA
Stl2-CR	agataagcgtcggaggaggaatttgatatacgagctacaacaagagcacg GAATTCGAGCTCGTTTAAAC
Hog1-check-F	tatctgccacgtttgatgacc
Hog1-check-R	ataagtgacggttcttggagtc
Slt2-check-F	agagtatgatggagatgagacctg
Slt2-check-R	ttattactgctactggatttccc
Slt2-LF	CGGGGTACCtatgtgaggtggacagtggg, KpnI site underlined
Slt2-LR	CggAATTCatcttgattgaagaccttgaaag, EcoRI site underlined
HAC1-RT-F	aggaaaaggaacagcgaagg
HAC1-RT-R	gaattcaaacctgactgcgc
