# Supplementary figures and images for: Roles of High Osmolarity Glycerol and Cell Wall Integrity Pathways in Cadmium Toxicity in Saccharomyces cerevisiae
Source: Int J Mol Sci. 2021 Jun 8;22(12):6169. doi: 10.3390/ijms22126169 (PMC8226467; doi:10.3390/ijms22126169)

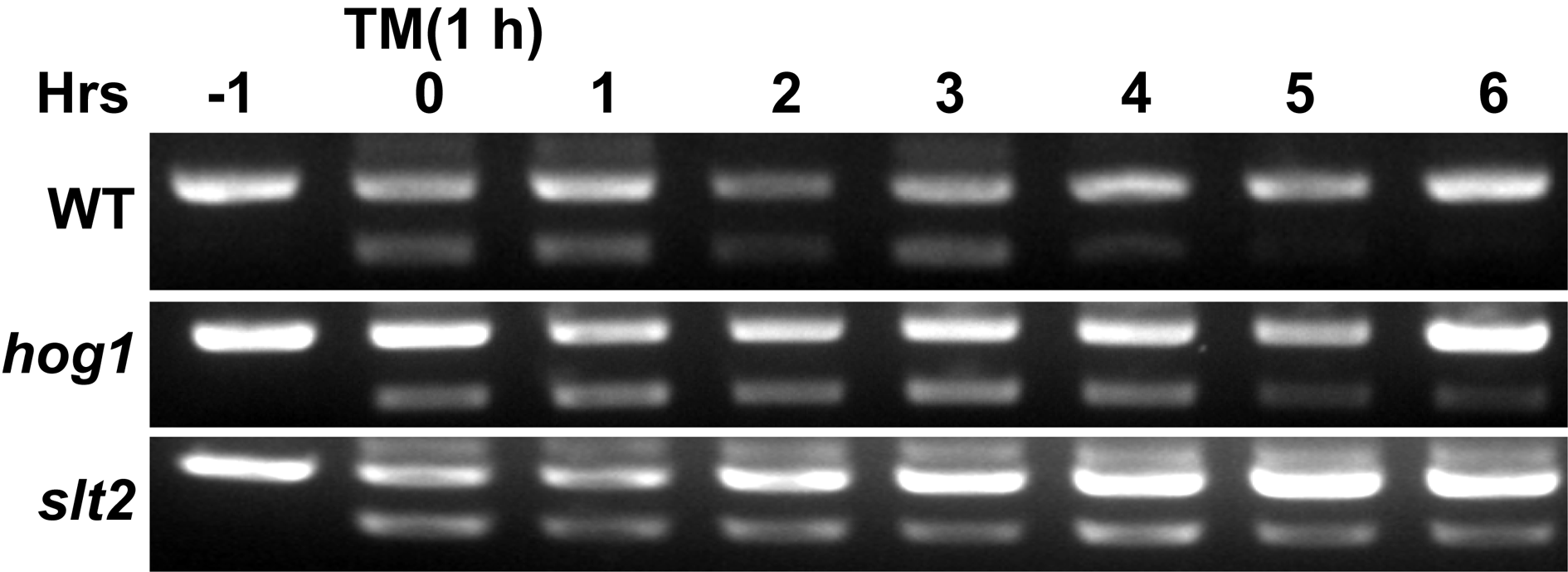

Supplement: Supplementary file 1 [file ijms-22-06169-s001.zip › ijms-1231017-SI/Figure S1.tif]
